# Astrocytes in Down Syndrome Across the Lifespan

**DOI:** 10.3389/fncel.2021.702685

**Published:** 2021-08-18

**Authors:** Blandine Ponroy Bally, Keith K. Murai

**Affiliations:** Centre for Research in Neuroscience, Department of Neurology and Neurosurgery, Brain Repair and Integrative Neuroscience Program, Research Institute of the McGill University Health Centre, Montreal General Hospital, Montreal, QC, Canada

**Keywords:** Down Syndrome, astrocyte, neurodevelopment, intellectual disability, glia, Alzheimer’s disease

## Abstract

Down Syndrome (DS) is the most common genetic cause of intellectual disability in which delays and impairments in brain development and function lead to neurological and cognitive phenotypes. Traditionally, a neurocentric approach, focusing on neurons and their connectivity, has been applied to understanding the mechanisms involved in DS brain pathophysiology with an emphasis on how triplication of chromosome 21 leads to alterations in neuronal survival and homeostasis, synaptogenesis, brain circuit development, and neurodegeneration. However, recent studies have drawn attention to the role of non-neuronal cells, especially astrocytes, in DS. Astrocytes comprise a large proportion of cells in the central nervous system (CNS) and are critical for brain development, homeostasis, and function. As triplication of chromosome 21 occurs in all cells in DS (with the exception of mosaic DS), a deeper understanding of the impact of trisomy 21 on astrocytes in DS pathophysiology is warranted and will likely be necessary for determining how specific brain alterations and neurological phenotypes emerge and progress in DS. Here, we review the current understanding of the role of astrocytes in DS, and discuss how specific perturbations in this cell type can impact the brain across the lifespan from early brain development to adult stages. Finally, we highlight how targeting, modifying, and/or correcting specific molecular pathways and properties of astrocytes in DS may provide an effective therapeutic direction given the important role of astrocytes in regulating brain development and function.

## General Features of the DS Brain

Down Syndrome (DS) is a genetic condition found in approximately one in 400 births and results from the presence of an extra copy of human chromosome 21 (Hattori et al., 2001). Trisomy 21 alters gene expression in all cells of the body and results in characteristic facial features, hypothyroidism, hearing and vision abnormalities, cardiac and gastric malformations, and importantly, delayed brain and cognitive development (Baburamani et al., 2019; Vicente et al., 2020). Neurodevelopment is atypical and extremely variable in DS. Notably DS individuals present intellectual disability ranging from mild to severe [30–70 of intellectual quotient (IQ)] (Maatta et al., 2006). Such intellectual disability manifests itself by disrupting working memory and verbal short-term memory (Chapman and Hesketh, 2000; Lanfranchi et al., 2012). Analyses of the DS brain and animal models have shown reduced brain volume, as well as, simplified gyral appearance in human samples (Pinter et al., 2001). Furthermore, in children with DS, the brain has reduced cortical area but an increased cortical thickness (Lee et al., 2016). Later, during early to middle adulthood, the DS brain shows signs of premature aging and shrinkage of crucial brain regions needed for learning and memory and executive function, such as the hippocampus and prefrontal cortex (Koran et al., 2014). During middle to late adulthood, essentially all DS individuals develop AD neuropathology, including ß-amyloid (Aß) plaques, tauopathy, neurodegeneration, and neuroinflammation (Wisniewski et al., 1978, 1985; Mann and Esiri, 1989).

The neurodevelopmental and intellectual deficits observed in DS are strongly linked to alterations in brain connectivity. Indeed, connectivity in the brain of DS individuals is reported to be perturbed at multiple levels. MRI studies have shown that DS individuals have altered functional connectivity and synchrony (Anderson et al., 2013; Pujol et al., 2015; Figueroa-Jimenez et al., 2021). However, disruptions in connectivity are not uniform across the brain, but rather occur in areas where anatomical alterations have been reported such as the hippocampus, anterior cingulate cortex (ACC), and the frontal lobe (Aylward et al., 1999; Carducci et al., 2013), and are consistent with the cognitive deficits observed with DS. Excessive connectivity along with increased inter-brain regional connectivity are thought to contribute to poor adaptative behaviors and lower IQ (Anderson et al., 2013; Pujol et al., 2015). At the cellular level, a reduction in neuronal production and premature neuronal death are observed in DS and implicated in the brain size reduction (Contestabile et al., 2007; Guidi et al., 2008, 2011). Abnormal dendritic arborization, dendritic spine density and morphology are also reported in DS (Takashima et al., 1981; Wisniewski et al., 1984; Becker et al., 1986), indicating disrupted formation and maintenance of cellular connectivity.

While neuronal changes have been widely described in DS, it is far less clear how trisomy 21 impacts non-neuronal cells which are essential for brain development, function, and homeostasis. Interestingly, recent studies analyzing the transcriptome of human DS brains have revealed a dysregulation of genes involved in oligodendrocyte differentiation, these genes include *TMEM63A*, *MYRF*, *PLD1*, *RTKN*, *ASPA*, *OPALIN*, *ERBB3*, and *EVI2A* (Olmos-Serrano et al., 2016). This is consistent with defects in axonal myelination and altered psychomotor development in DS individuals (Wisniewski and Schmidt-Sidor, 1989). Alterations of oligodendrocytes are also present in the Ts65dn mouse model [the most studied DS mouse model which consists of a partial trisomy made up of a distal portion of mouse chromosome 16 and a centromeric portion of mouse chromosome 17 (Davisson et al., 1993)], where defects in myelin are attributed to impairments in oligodendrocyte maturation and an overall reduction in the number of mature myelinating oligodendrocytes (Olmos-Serrano et al., 2016). Thus, trisomy 21 appears to impact non-neuronal cells including oligodendrocytes, an important brain cell type that ensures the fidelity of axon potential conduction.

## Astrocytes in Neurodevelopmental Disorders and Neurodegenerative Diseases

In addition to oligodendrocytes, astrocytes are a highly abundant non-neuronal types in the central nervous system (CNS) (Herculano-Houzel, 2014) that play central roles in the healthy and diseased brain at all stages of life. Astrocytes were originally thought to create passive connective tissue within the brain (Volterra and Meldolesi, 2005). However, we now know that they are critical for numerous aspects of brain function and possess sophisticated mechanisms to communicate with other CNS cell types, especially neurons. Astrocytes provide important metabolic and neurotrophic support to neurons (Rouach et al., 2008; Belanger and Magistretti, 2009; Figley, 2011; Dezonne et al., 2013) and regulate key processes such as synapse formation/plasticity, extracellular ion/neurotransmitter homeostasis, and neurovascular coupling (Drejer et al., 1982; Denis-Donini et al., 1984; Parpura et al., 1994; Zhang and Barres, 2010; Farmer and Murai, 2017; Matias et al., 2019), thus making them essential regulators of neurons across the lifespan. During development, astrocytes make important contributions to axon guidance and synapse formation and plasticity. Thus, it is not surprising that astrocytes have been implicated in neurodevelopmental disorders including Rett (RTT) and Fragile X (FXS) syndromes and autism spectrum disorders (ASD). Such disorders are characterized by abnormalities in neuronal brain wiring and physiology, with many studies focused on disruptions to neurogenesis, axon guidance, dendrite development, and synaptogenesis. Recent studies have suggested that astrocytes may directly participate in such disorders. For example, increased levels of several glial proteins have been identified including GFAP, EAAT1, and S100A3 in post mortem brain samples from RTT individuals. RTT also causes astrocyte reactivity (Colantuoni et al., 2001), in which the structural, molecular, and functional profile of astrocytes is significantly altered (Escartin et al., 2021). *In vitro*, RTT astrocytes release factors into the culture medium which stunt neurite outgrowth (Ballas et al., 2009). Astrocytes also are implicated in FXS where dendritic arborization is delayed in neurons co-cultured with FXS astrocytes (Jacobs et al., 2010). *In vivo* studies in mice have also shown that selective genetic deletion of Fmr1 (gene responsible for FXS) from astrocytes reduces spine dynamics and impairs motor-skill learning (Hodges et al., 2017). Astrocytic alterations have been shown in non-syndromic ASD using human tissues and animals models and has been reviewed recently (Petrelli et al., 2016). Interestingly, the astrocytic glutamate transporters EAAT1 and EAAT2, which regulate extracellular glutamate levels at the synapse, are misregulated in the brain of ASD individuals (Purcell et al., 2001). The importance of astrocytes in ASD has recently been further supported by transcriptome analysis of the ASD brain (Voineagu et al., 2011). These studies revealed an enrichment of reactive astrocytes (Voineagu et al., 2011) which is supported by immunolabeling of post mortem ASD brain tissue where reactive gliosis and glial proliferation are observed (Vargas et al., 2005; Edmonson et al., 2014). While much has been learned about the contribution of astrocytes to neurodevelopmental disorders such as RTT, FXS, and ASD [which is further described in this review (Cresto et al., 2019)], further work is still required to pinpoint precisely how these cells contribute to both morphological and functional changes of the developing brain.

Beyond neurodevelopmental disorders, astrocytes also participate in the pathological events following acquired CNS injuries such as stroke, spinal cord injury, and traumatic brain injury, where robust astrocyte reactivity is found near sites of injury (Anderson et al., 2016; Burda et al., 2016; Adams and Gallo, 2018). Astrocyte reactivity is also a prominent feature in neurodegenerative conditions, such as Alzheimer’s disease (AD), Parkinson’s disease (PD), multiple sclerosis (MS), and amyotrophic lateral sclerosis (ALS) (Allan and Rothwell, 2003; Belanger and Magistretti, 2009; Sofroniew and Vinters, 2010). The role of astrocytes in these conditions is described in detail in several reviews (Sofroniew and Vinters, 2010; Pekny et al., 2016; Verkhratsky et al., 2016). With both acquired CNS injuries and chronic neurodegenerative conditions, astrocytes significantly change their communication with neurons and microglial cells, the residing immune cells of the CNS, and participate in a variety of non-cell autonomous processes through the production of neuro-modulatory and inflammatory mediators (such as cytokines), and growth factors. This gives rise to a complex scenario where astrocytes can have both neuroprotective and neurotoxic effects that are context-dependent (Burda and Sofroniew, 2014; Hong et al., 2016; Kwon and Koh, 2020). Further discussion about astrocyte reactivity will come later in this review as it is a prominent feature of the DS brain and akin to what is seen in other neurodegenerative diseases, especially AD.

## DS Astrocytes and Brain Size

**FIGURE 1:**
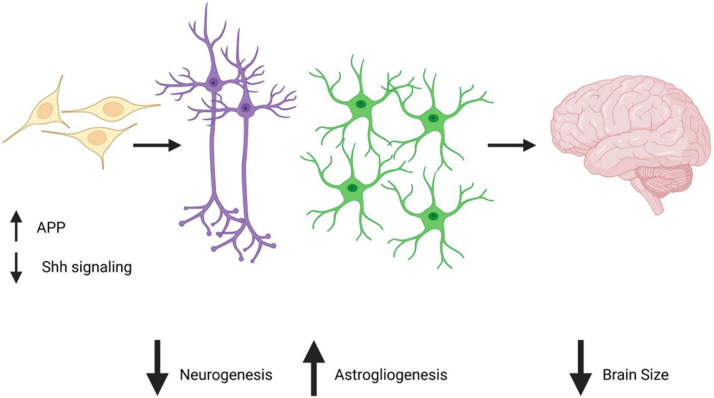
APP overexpression in neuroprogenitor cells decreases Shh signaling and is believed to be responsible for an increase in astrogliogenesis and a decrease in neurogenesis. Created with Biorender.com.

As astrocytes play important roles in brain disorders and diseases from early developmental to adult stages, it is important to consider how astrocytes are affected by triplication of chromosome 21. Although studies of astrocytes in DS are few in number when compared to studies of neurons, recent studies are providing insight into how trisomy 21 can directly impact astrocytes to affect brain development and function throughout the lifespan. A characteristic feature of the DS brain is reduced brain volume which likely contributes to the intellectual disability of DS individuals. This reduced brain volume has been reported as early as in the second trimester of pregnancy (Patkee et al., 2020) and is caused by a significant reduction in neuronal number (Schmidt-Sidor et al., 1990). It should be noted that cell counting in the brain is not an easy task and can be associated with significant analytical artifact which is described in this review (von Bartheld et al., 2016). Therefore, additional and up to date studies performed in humans would be useful in order to calculate more precise numbers and ratios of astrocytes and neurons in DS. However, considering the large body of evidence reporting smaller brain volumes and decreased neuronal numbers in humans and animal models from *in vivo* and *in vitro* analysis, it is largely accepted that this phenomenon occurs in the DS brain. New studies using contemporary techniques would nevertheless allow for clarity and preciseness in the exact numbers and ratios. It is suspected that there are multiple causes of the reduction in neuronal number, including a decrease in neuronal differentiation during development and increased neuronal cell death throughout the life of DS individuals (Contestabile et al., 2007; Guidi et al., 2008, 2011). Notably, the reduction in neuronal differentiation is believed to be caused by a gliogenic shift, meaning that neuroprogenitor cells alter their differentiation ability in favor of astrocytes rather than neurons (Guidi et al., 2008; Zdaniuk et al., 2011; Chen et al., 2014). Several mechanisms can cause the gliogenic shift in DS, among which is a decrease in progenitor cell proliferation (Roper et al., 2006; Contestabile et al., 2007; Trazzi et al., 2013) and deficits in the Sonic hedgehog signaling pathway which have been directly shown to cause a reduction in the production of neurons (Roper et al., 2006; Currier et al., 2012; Das et al., 2013; Trazzi et al., 2013). Remarkably, studies have shown that correcting these deficits can rescue neuronal number in a DS animal model (Das et al., 2013). Indeed, with a single injection of a Sonic hedgehog agonist in newborn mice, the Reeves’ group restored neuronal number as well as behavioral deficits in the DS model. Furthermore, studies performed *in vitro* demonstrated that excessive levels of AICD (amyloid intracellular domain), which results from the cleavage of APP by γ-secretase were responsible for the increase in expression of Ptch1 and therefore for the malfunctioning of the Sonic hedgehog pathway. Importantly this study also showed that the treatment of neuronal precursor cells with a γ-secretase inhibitor normalized AICD and restored neurogenesis and gliogenesis levels to normal levels *in vitro* (Figure 1; Giacomini et al., 2015). Additional pathways have also been suggested to induce a gliogenic shift in DS, such as an increase in progenitor cell oxidative stress and apoptosis caused by the simultaneous overexpression of S100ß and amyloid precursor protein (APP) (both of which are genes located on chromosome 21) (Lu et al., 2011). Finally, overactivation of the JAK-Stat pathway in progenitor cells due to Dyrk1a overexpression (also a chromosome 21 gene) has been suggested to drive aberrant gliogenesis in DS (Kurabayashi et al., 2015; Lee et al., 2019). Overall, the gliogenic shift in DS and the pathways described could potentially be targeted to rescue neuronal number deficits and restore cell populations that are competent to form normal brain cell number and connectivity.

## DS Astrocytes and the Development of Brain Connectivity

**FIGURE 2:**
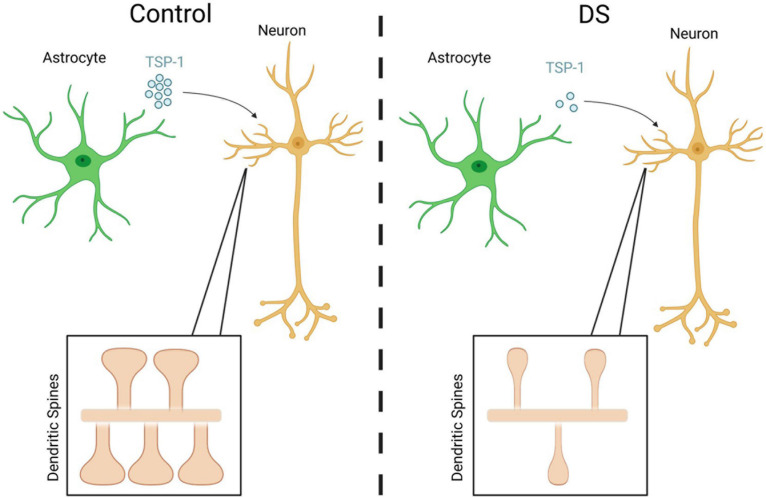
Reductions in the astrocytic production and secretion of TSP-1 from DS cells causes abnormal dendritic spine shape and number *in vitro*. Created with Biorender.com.

In DS, neuronal connectivity is believed to be disrupted at several levels from individual synapses to whole circuits. Alterations in synapse density and shape have been reported in the brains of DS individuals, along with defects in dendritic outgrowth and arborization (Marin-Padilla, 1976; Coyle et al., 1986; Ferrer and Gullotta, 1990; Golden and Hyman, 1994; Benavides-Piccione et al., 2004). The extensiveness of such morphological abnormalities are correlated with the severity of intellectual disability (Zdaniuk et al., 2011). Interestingly, dendritic arborization and synaptogenesis are processes which are regulated by astrocytes in the developing brain through the expression and/or release of various neuroactive factors (Mauch et al., 2001; Christopherson et al., 2005; Verkhratsky and Butt, 2007). Interestingly, thrombospondin 1 (TSP-1), a known astrocyte-secreted synaptogenic factor (Christopherson et al., 2005), is expressed at lower levels in cultured DS astrocytes (Garcia et al., 2010). Its lowered expression is responsible for perturbations in dendritic spine morphology and decreases in synapse number in co-cultures of human DS astrocytes with rodent neurons (Figure 2). This deficit can be mitigated by supplementation with recombinant TSP-1 (Garcia et al., 2010). Future experiments, such as those investigating TSP-1 in DS animal models, will be important in assessing how lower TSP-1 levels contribute to DS synaptic changes that impact neuronal connectivity and circuit formation, and cognitive processes such as learning and memory formation.

**FIGURE 3:**
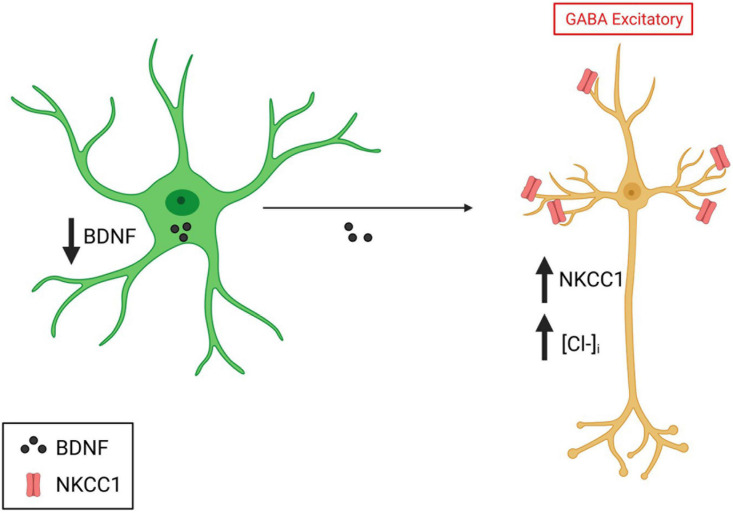
Implication of astrocytes in the GABA switch in DS. Created with Biorender.com.

In addition to defects in synapse development, perturbations in the effectiveness of GABA synaptic transmission are also implicated in DS (Contestabile et al., 2017). GABA is the main inhibitory neurotransmitter in the mature brain. However, during development, GABA transmission is known to be excitatory (Ben-Ari, 2002). During the postnatal period, GABAergic responses in neurons switch from being excitatory to inhibitory due to decreases and increases in the expression of the chloride transporters NKCC1 and KCC2, respectively, which regulate intracellular chloride concentration (Ben-Ari, 2002). In DS, inhibitory GABA transmission in the adult brain is altered and rendered excitatory. This is supported by studies in the Ts65dn mouse model that have shown that synaptic plasticity and memory deficits can be corrected when inhibitory GABA transmission is restored (Deidda et al., 2015). Intriguingly, astrocytes can regulate intracellular GABA concentrations and the GABA excitatory-inhibitory switch *in vitro* (Li et al., 1998). This is mainly through the secretion of BDNF, which downregulates NKCC1 levels (Eftekhari et al., 2014). Studies have demonstrated that both BDNF and NKCC1 levels are altered in DS (Deidda et al., 2015). Indeed the reduction in BDNF levels (Tlili et al., 2012) and upregulation of NKCC1 cause excitatory GABAergic transmission in the adult brain (Figure 3; Deidda et al., 2015). Thus, astrocytes may alter the GABA switch in the DS brain through their altered BDNF secretion and cause cellular and circuit-level deficits in excitation/inhibition in the developing brain.

Unlike neurons, astrocytes do not have action potentials. Instead, they exhibit dynamic physiological changes visualized through intracellular calcium elevations. Such communication is coordinated by intracellular calcium transients which can be driven by neuronal activity (Khakh and McCarthy, 2015). These calcium events are thought to induce release of neuroactive molecules including gliotransmitters which can alter neuronal activity and the activity of neighboring astrocytes (Angulo et al., 2004; Lee et al., 2010). In DS, aberrant calcium dynamics have been reported both in rodent models (Muller et al., 1997) and in one study using induced pluripotent stem cell (iPSC)-derived human astrocytes in which spontaneous calcium fluctuations were increased (Mizuno et al., 2018). These aberrant calcium dynamics are believed to cause a reduction in neuronal excitability when co-cultured with neurons. Remarkably, S100ß overexpression causes aberrant calcium signaling, and pharmacological intervention on this pathway restores calcium dynamics along with neuronal excitability (Mizuno et al., 2018). Thus, targeting calcium dynamics in DS astrocytes may improve aberrant neuronal activity patterns in DS. However, a recent study performed by our group did not detect similar alterations in spontaneous or evoked calcium fluctuations in three different iPSC-derived DS astrocyte lines (Ponroy Bally et al., 2020). The reason for this discrepancy is unclear. However, it should be noted that the two studies were performed using different iPSC (Figure 5) cell lines and that in Mizuno et al. (2018), only one isogenic line was used. Therefore, additional studies are required using a larger number of iPSC lines in order to better understand the impact of trisomy 21 on astrocyte calcium dynamics.

**FIGURE 4:**
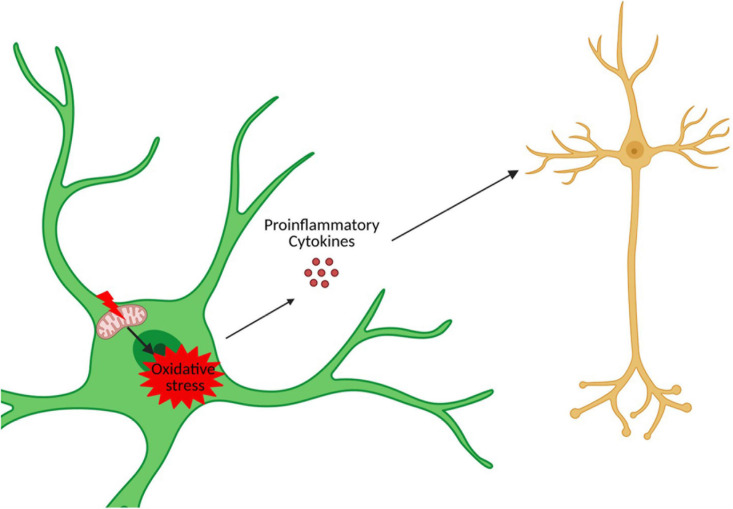
Astrocytic oxidative stress and mitochondrial dysfunction in neuronal death. Created with Biorender.com.

**FIGURE 5:**
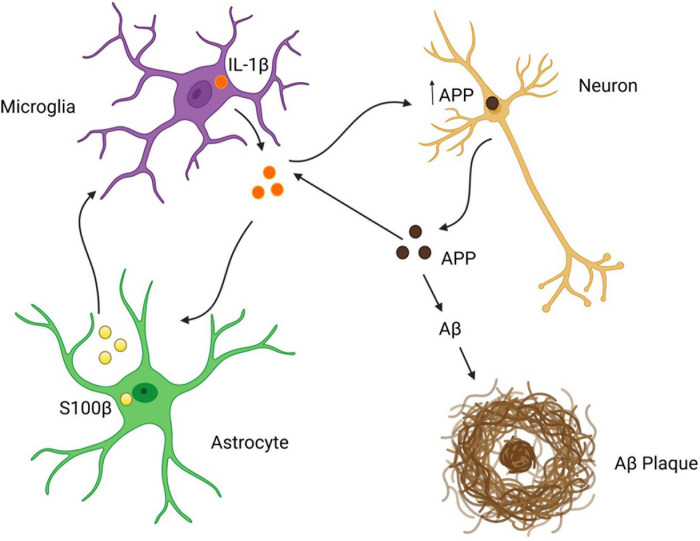
Astrocytes as drivers of Aß pathology in the DS brain? Created with Biorender.com.

## Genome-Wide Transcriptional Alterations in DS Astrocytes

Until recently, it was believed that the DS phenotype was largely caused by the altered gene dosage of a small number of genes located in the DS critical region (DSCR) of chromosome 21 (Korenberg, 1990; Delabar et al., 1993). The DSCR extends for approximately 5.4 Mb on HSA21q22 and was shown to be necessary and sufficient to induce a DS phenotype. Many key genes are in that region such as DYRK1A, APP, S100β and SOD1 (Belichenko et al., 2009). However, studies now show that trisomy 21 has broader and more complex effects well beyond those directly associated with the DSCR (Korbel et al., 2009; Lyle et al., 2009). A recent study reported changes to the global chromatin architecture in DS, with potential genome-wide effects on the transcriptome (Letourneau et al., 2014). Other studies have reported global transcriptional alterations in DS from analysis of a range of tissues including brain (Saran et al., 2003; Mao et al., 2005; Lockstone et al., 2007; Olmos-Serrano et al., 2016), heart (Mao et al., 2005), blood (Pelleri et al., 2018), and thymus (Pelleri et al., 2018), as well as, in individual cell types including fibroblasts (Pelleri et al., 2018), fetal cells (FitzPatrick et al., 2002), lymphoblastoid cell lines (Sullivan et al., 2016), iPSCs (Briggs et al., 2013; Weick et al., 2013; Gonzales et al., 2018; Pelleri et al., 2018), and neurons (Briggs et al., 2013; Weick et al., 2013; Gonzales et al., 2018; Huo et al., 2018). With respect to astrocytes, microarray analysis has detected dysregulation of many mRNAs in these cells (Chen et al., 2014). A more recent study from our group using an Assay for Transposase Accessible Chromatin sequencing (ATAC-seq) on control and DS iPSC-derived astrocytes uncovered thousands of differently accessible chromatin sites across the genome in DS astrocytes, with an even split of increased and decreased accessibility (Ponroy Bally et al., 2020). Concomitantly, RNA sequencing (RNA-seq) revealed a global dysregulation of the transcriptome of DS astrocytes that differed significantly from DS neuroprogenitors (Ponroy Bally et al., 2020). As expected, DS astrocytes showed an upregulation of genes on chromosome 21 such as DYRK1A, S100β, APP, SOD1 and SUMO3. However, 93% of dysregulated genes were found outside of chromosome 21 and were distributed across the genome. Interestingly, mRNAs encoding cell adhesion and extracellular matrix (ECM)-related genes were especially altered and led to impaired adhesive properties of these cells. This is particularly interesting as alterations in cell adhesion and the ECM have also been reported in various cell types and tissues in DS (Conti et al., 2007; Gonzales et al., 2018; Huo et al., 2018). Further investigation into cell adhesion changes of astrocytes is needed to better understand their relationship to neurodevelopmental and age-related changes observed in the DS brain.

## DS Astrocytes and Neuronal Injury

Trisomy 21 is expected to impact astrocyte physiology throughout the lifespan of DS individuals. Mitochondrial dysfunction and oxidative stress may be particularly relevant in this context given that these processes are associated with DS and are a common feature of all DS cells and tissues including astrocytes (Izzo et al., 2018). Consistent with this, DS astrocytes contain a fragmented mitochondrial network that is composed of mostly shorter mitochondria and few elongated mitochondria (Helguera et al., 2013). This type of mitochondrial network is correlated with reduced ATP production and increased ROS production (Yu et al., 2006). This lowered mitochondrial activity in DS astrocytes may be an adaptative and protective mechanism. Indeed, DS astrocytes are able to increase their mitochondrial activity if stimulated, but this exacerbates free radical formation, lipid peroxidation, and cell death (Helguera et al., 2013). Consistent with this, increased oxidative stress has been reported in various DS cell types, including astrocytes, and increases in iNOS and nitrite/nitrate concentrations have been reported in the conditioned medium of iPSC-derived astrocytes. Importantly, an increase in astrocytic oxidative stress can cause an increase in neuronal cell death (Hu et al., 1997; Chen et al., 2014). Thus, mitochondrial dysfunction and oxidative stress in DS astrocytes may impact the health of neurons and contribute to neuronal cell death observed within the DS brain (Figure 4).

## Astrocytes and Alzheimer’s Disease Pathology in DS

Improvements in health care systems and management of co-morbidities in DS have led to a dramatic increase in life expectancy for DS individuals from 12 years of age in 1949 to 60 years of age in 2004 (Bittles and Glasson, 2004). This increase in life expectancy has also led to the discovery of age-related conditions in DS, the main one being AD neuropathology. Indeed, by age 40, most (if not all) DS individuals present AD neuropathology including Aß plaques, neurofibrillary tangles, neurodegeneration, and neuroinflammation (Mann, 1988; Mann and Esiri, 1989; Motte and Williams, 1989; Zigman and Lott, 2007). The prevalence of dementia in DS-associated AD is similar to sporadic AD (Oliver et al., 1998), although evaluating the cognitive decline in DS is challenging due to the pre-existing intellectual impairment. Novel cognitive tests are currently being developed and deployed to better assess the abilities of DS individuals (Sinai et al., 2016; Dekker et al., 2018). Importantly, dementia is associated with the mortality of over 70% of DS individuals making it the main cause of death in DS (Hithersay et al., 2018). In familial AD, ∼10% of cases are caused by mutations in the APP protein, which is a precursor to toxic Aβ in plaques. The APP gene is found on chromosome 21 and its overexpression is thought to be a primary cause of AD in DS leading to a rapid accumulation of Aβ with age (Margallo-Lana et al., 2004; Head et al., 2016).

In AD, chronic neuroinflammation is believed to exacerbate Aβ burden and possibly neurofibrillary tangle formation related to tau hyperphosphorylation, thus potentially linking two hallmarks of AD pathology (Kinney et al., 2018). In DS, astrocyte reactivity may be a major contributor to AD pathology given that glial reactivity has been reported to occur as early as 2 days postnatally (Griffin et al., 1989). This early astrocytic reactivity occurs prior to wide-spread Aß plaque formation and neuronal degeneration, and hence is in a position to play a primary role in AD pathology. Consistent with this, early overexpression of S100ß in astrocytes and neuronal APP overexpression have been shown to activate microglia and increase IL-1β expression which, in turn, which exacerbates APP production in neurons and glial cells (Goldgaber et al., 1989; Barger and Harmon, 1997; Liu et al., 2005). These events appear to be self-propagating, as IL-1β and S100ß have both been reported to induce microglial cell activation and astrocyte reactivity with overexpression of themselves, as well as, neuronal APP (Goldgaber et al., 1989; Sheng et al., 1996). Notably, glial activation and cytokine production occur during childhood in DS, many years before the accumulation of Aβ plaques (Griffin et al., 1989). Taken together, upregulation of neuronal APP and astrocytic S100ß, and cytokines such as IL-1ß, may drive neuronal stress, glial activation, and DS-related neuropathological changes characteristic of AD (Figure 5).

A noticeable cellular feature accompanying AD pathology is oxidative stress. Increased oxidative stress and ROS production occur in various cell types in DS throughout the lifespan including in astrocytes. Increased ROS production in the aging DS brain is known to damage proteins, lipids, and DNA which alters neuronal function and ultimately aggravates neurodegeneration in DS (Busciglio and Yankner, 1995; Perluigi and Butterfield, 2012; Di Domenico et al., 2013; Perluigi et al., 2014a). Studies have demonstrated that increased ROS levels in neurons leads to altered processing of APP and accumulation of Aβ (Busciglio et al., 2002; Cenini et al., 2012; Coskun and Busciglio, 2012; Perluigi and Butterfield, 2012). Interestingly, the spread and extent of oxidative stress increases with age and correlates with Aβ levels (Lott et al., 2006). The progressive and chronically high level of oxidative stress is therefore implicated in neuronal death and believed to contribute to neurodegenerative processes and cognitive dysfunction in the DS brain (Perluigi and Butterfield, 2012). Correcting oxidative stress early in DS may help ameliorate premature aging and slow the progression of AD neuropathology with which there are no therapies. Consumption of vitamin-rich diets and vitamin supplementation may also combat neurodegeneration, since vitamins are known antioxidants and reduce oxidative stress (Bhatti et al., 2016). Thus, targeting oxidative stress pathways may hold promise for the future treatment of DS-associated AD.

Excitotoxicity is another major event which lead to neuronal death and neurodegeneration and may be related to AD-related neuropathology in DS. Astrocyte reactivity has been shown to exacerbate excitotoxicity and neurodegeneration through the overexpression of the metabotropic receptor mGluR5. mGluR5 is expressed in both astrocytes and neurons and is important for neuron-glial cell communication in both the healthy and injured brain (Aronica et al., 2003; D’Antoni et al., 2008; Bradley and Challiss, 2012). mGluR5 is prevalent in the developing brain and is involved in processes such as proliferation, differentiation, and survival of neuronal progenitors (Di Giorgi Gerevini et al., 2004). mGluR5 expression is lower in the adult brain, except in areas with active neurogenesis (Catania et al., 1994; Romano et al., 1996). Importantly, mGluR5 upregulation has been reported in brains of AD and DS individuals (Oka and Takashima, 1999; Dolen and Bear, 2008; Kumar et al., 2015). This mGluR5 upregulation is specific to astrocytes in DS and occurs as early as mid-gestation and persists postnatally (Iyer et al., 2014b). Aged DS individuals with AD pathology also present even higher levels of mGluR5 in astrocytes, especially in astrocytes in close vicinity to Aβ plaques. This suggests that Aβ may stimulate upregulation of mGluR5 expression in astrocytes (Iyer et al., 2014a). Interestingly, astrocytic mGluR5 is activated by soluble Aβ in sporadic AD which has been found to generate calcium oscillations and the release of glutamate, thus enhancing neuronal excitotoxicity (Shrivastava et al., 2013; Kumar et al., 2015).

The mammalian target of rapamycin (mTOR) pathway is also known to be altered in DS astrocytes. The mTOR pathway is an important signaling pathway which responds to a large variety of environmental stimuli and regulates essential processes such as cell growth and proliferation, metabolism, protein synthesis, synaptogenesis, and apoptosis (Dazert and Hall, 2011; Laplante and Sabatini, 2012; Wong, 2013). Dysregulation of this pathway has a major impact on the nervous system and has been reported to occur in various neurological diseases such as tuberous sclerosis (Orlova and Crino, 2010), ASD (Tsai et al., 2012), and DS. This pathway has also been identified as a molecular link between Aβ accumulation and cognitive dysfunction in sporadic AD. Intriguingly, mTOR inhibitors can reverse cognitive dysfunction and reduce Aβ load in a mouse model of AD (Caccamo et al., 2010; Ma et al., 2010; Spilman et al., 2010) and hyperactivation of the mTOR pathway has been identified both in the developing and aged DS brain (Iyer et al., 2014a; Perluigi et al., 2014b). A recent study showed that iPSC-derived DS astrocytes cause mTOR hyperactivation in control neurons and exacerbate the hyperactivation in DS neurons (Araujo et al., 2018). Targeting mTOR hyperactivation in astrocytes and neurons may therefore be a plausible target for mitigating some aspects of AD pathology in DS.

## Final Perspective About Astrocytes and DS

New discoveries are challenging the neurocentric view of DS and leading to a more complete understanding of the contributions of other brain cell types including astrocytes to DS pathophysiology. Recent studies have revealed myriad ways astrocytes can participate in DS across the lifespan. Although these studies still remain relatively few in number, they provide an important launching point for investigating how trisomy 21 alters their properties which may have profound effects on the developing and aging DS brain. Since astrocyte development largely occurs postnatally, there may be an attractive therapeutic window for correcting genetic or molecular alterations in DS to improve brain function and prevent cellular changes including AD-related neuropathology. Harnessing new technologies such as single cell RNA-seq to investigate transcriptional profiles and cellular heterogeneity in DS will allow additional detailed characterization of astrocytes in the DS brain. This technology has been used in other diseases such as in Huntington’s disease where several transcriptional states of astrocytes were identified (Al-Dalahmah et al., 2020). Use of patient-derived iPSCs is also a relatively new technology allowing the study of human DS cells. This approach is compatible with high-throughput screening methods that can be used to identify new compounds that correct aberrant cellular pathways caused by trisomy 21. However, a current limitation of iPSC research is the limited availability of independent DS cell lines. Many studies have used the same iPSC lines, and it is clear that genetic background can have an important impact on cellular phenotypes observed, especially in DS. New patient-derived iPSC lines need to be created and shared among the scientific community in order to take full advantage of this powerful approach. Finally, establishing new animal models of DS is an important future direction for the field to help validate findings *in vivo* and test new hypotheses. DS is a particularly challenging to model in mice as it requires the triplication of genes of a whole human chromosome which are spread over several chromosomes in mice. There are many different animal models of DS which all have their strengths and weaknesses and summarized in this review (Herault et al., 2017). Access to new models and application of innovative technologies such as single-cell RNA-seq and iPSCs will help build a more complete picture of the cellular changes occurring in DS and provide further optimism that effective therapies for DS can be found.

## Author Contributions

BPB and KKM wrote the manuscript. Both authors contributed to the article and approved the submitted version.

## Conflict of Interest

The authors declare that the research was conducted in the absence of any commercial or financial relationships that could be construed as a potential conflict of interest.

## Publisher’s Note

All claims expressed in this article are solely those of the authors and do not necessarily represent those of their affiliated organizations, or those of the publisher, the editors and the reviewers. Any product that may be evaluated in this article, or claim that may be made by its manufacturer, is not guaranteed or endorsed by the publisher.
